# Risk Factors of Infections Due to Multidrug-Resistant Gram-Negative Bacteria in a Community Hospital in Rural Thailand

**DOI:** 10.3390/tropicalmed7110328

**Published:** 2022-10-23

**Authors:** Jindanoot Ponyon, Anusak Kerdsin, Thanawadee Preeprem, Ratchadaporn Ungcharoen

**Affiliations:** 1 Faculty of Public Health, Chalermphrakiat Sakon Nakhon Campus, Kasetsart University, Sakon Nakhon 47000, Thailand; 2 Faculty of Pharmaceutical Sciences, Ubon Ratchathani University, Ubon Ratchathani 34190, Thailand

**Keywords:** risk factors, Gram-negative bacteria, multidrug resistance, logistic regression

## Abstract

Antimicrobial resistance is a major public health concern globally. The most serious antimicrobial resistance problem among pathogenic bacteria is multidrug resistance (MDR). The objectives of this study were to investigate the risk factors of MDR infections and to develop a risk assessment tool for MDR Gram-negative bacteria (MDR-GNB) infections at a community hospital in rural Thailand. The study revealed 30.77% MDR-GNB among GNB strains. The most common MDR-GNB strains were 63.02% for *Escherichia coli* and 11.46% for *Klebsiella pneumoniae*. A case–control study was applied to collect clinical data between January 2016 and December 2020. Univariate logistic regression and multivariate logistic regression were used to analyze the risk factors for MDR-GNB and a risk assessment score for each factor was determined based on its regression coefficient. The risk factors for MDR-GNB infections were as follows: the presence of Enterobacteriaceae that produce extended-spectrum beta-lactamase (ESBL) (OR_Adj._ 23.53, 95% CI 7.00–79.09), infections occurring within the urinary tract (OR_Adj._ 2.25, 95% CI 1.44–3.53), and patients with a history of steroid usage (OR_Adj._ 1.91, 95% CI 1.15–3.19). Based on the assigned risk scores for each associated factor, the newly developed risk assessment tool for MDR-GNB infections achieved 64.54% prediction accuracy (AUC-ROC 0.65, 95% CI 0.61–0.68), demonstrating that the tool could be used to assess bacterial infection cases in community hospitals. Its use should provide practical guidance on MDR evaluation and prevention. This study was part of an antibiotic stewardship program; the study surveyed antibiotic-resistant situations in a hospital and implemented an effective risk assessment tool using key risk factors of MDR-GNB infections.

## 1. Introduction

Antimicrobial resistance is a long-standing public health problem and is still a major global issue today [1,2,3]. The World Health Organization has estimated an increase in the number of deaths due to antimicrobial resistance from 700,000 annually in 2014 to 10 million annually by 2050. The number of deaths in Asian countries is expected to be as high as 4.7 million in 2050 [4]. In the United States, 2.8 million people have antibiotic-resistant infections each year, causing 35,000 deaths annually [5]. In Thailand, retrospective data from 2011 to 2012 showed that 111,295 people were infected with antimicrobial-resistant organisms, causing 48,258 deaths and 123,000 more days of hospitalization [6]. If there were a measure to prevent these patients from developing antimicrobial-resistant infections, the country’s medical expenses would be reduced by approximately THB 72 billion, including THB 8.3 billion toward antimicrobial medications. Considering the cost of illness from a societal perspective, the total cost coming from the patient and family expenses toward antimicrobial resistance is approximately THB 134.5 billion [6].

Gram-negative bacteria (GNB) that lead to problems of drug resistance differ between settings [1,3]. In the community, *Escherichia coli (E. coli)* and *Klebsiella pneumoniae (K. pneumoniae)* create a critical problem [7]. Major drug-resistant GNBs often found in inpatient wards include *Acinetobacter* spp., *Pseudomonas aeruginosa*, and carbapenem-resistant Enterobacteriaceae. Enterobacteriaceae that can produce extended-spectrum beta-lactamase (ESBL), such as *E. coli* and *K. pneumoniae*, are a major concern in humans, animals, and the environment [8].

Often, other studies on antimicrobial resistance have focused on risk factors of highly antimicrobial-resistant strains, analyzed only isolates of a specific micro-organism or isolates associated with some medications, or conducted the study in higher-level hospitals (general hospitals or hospital centers) [9,10,11,12,13]. Only a few studies have been performed in community hospitals [14,15,16]. In rural hospitals in Thailand, a microbial culture and antimicrobial susceptibility testing (AST) take a long time and, therefore, hinder the prompt classifications of patients at risk for antibiotic resistance. Hospitals should be established to tackle this problem. Therefore, the current study aimed to determine the risk factors for multidrug-resistant Gram-negative bacteria (MDR-GNB) and to develop a predictive model to assess the probability of MDR-GNB infections in hospitalized patients. The results could be applied toward prevention of antimicrobial resistance, promotion of appropriate antimicrobial use, development of a treatment guideline for drug-resistant infections, or prevention of microbial outbreaks in local community.

## 2. Materials and Methods

### 2.1. Study Design and Setting

This study was conducted at The Thatphanom Crown Prince Hospital, a middle-size community hospital (120 beds) located in the northeastern region of Thailand. The hospital has specialized physicians in five disciplines (internal medicine, pediatrics, obstetrics and gynecology, orthopedics, and surgery), making it a referral hospital for three lower-level hospitals nearby. The hospital provides inpatient services in five divisions, namely the pediatric ward, male ward, female ward, special ward, and intensive care unit. The wards are not assigned according to physicians’ specialties due to administrative purposes; therefore, antimicrobial resistance must be monitored in all wards due to the lack of patient classification.

A case–control study was conducted using past medical records between January 2016 and December 2020 at a community hospital in Nakhon Phanom province in northeastern Thailand. Cases and controls with a ratio of 1:2 were used in the study. The calculation of sample size was carried out using the STATA program (version 17), providing the odds ratio (OR) of antibiotic treatments in the last 3 months identified from a previous MDR-GNB study [17]. A two-sided test statistic was determined with a 90% predictive power at the β level of 0.10, an α significance level of 0.05, and the proportion of cases versus the control of 1:2. The calculation indicated satisfactory sample sizes of 98 and 196 for the cases and control, respectively. 

Cases were patients whose blood samples were positive for MDR-GNB, whereas controls were patients whose blood samples were positive for GNB but with only one or two types of antibiotic resistance, or with no drug resistance. 

#### 2.1.1. Inclusion Criteria

The inclusion criteria for this study were that the patients must have been admitted to the Thatphanom Crown Prince Hospital for more than 24 h, have had positive results on microbial cultures, and undergone antimicrobial susceptibility testing (AST). When a patient received multiple reports on microbial culture or AST, only the first report of each test was included in the analysis per patient. If a patient received antibiotic therapy in other institutions, it is not possible to obtain such medical records. Therefore, the analysis only included medical records owned by the Thatphanom Crown Prince Hospital. 

#### 2.1.2. Exclusion Criteria

Patients with incomplete information on biological and geographic data or with a lack of medical history in the Thatphanom Crown Prince Hospital within the specified period were initially excluded from this study. In addition, patients who stayed in the Thatphanom Crown Prince Hospital for less than 24 h were not selected for analysis.

### 2.2. Data Collection and Definition

The current study collected results of bacterial identification and AST from all patients who had been admitted to the Thatphanom Crown Prince Hospital between January 2016 and December 2020. Microbial laboratory analysis was conducted at the provincial hospital and the results were deposited into the MLAB database (version 5.58.22.02.28). Later, a list of designated patients was created and data on various factors to be studied were collected from their medical records and electronic medical records, with the latter stored in the HOSxP program.

According to the guidelines on the interpretation of antimicrobial susceptibility by the Clinical and Laboratory Standards Institute (CLSI), susceptibility was reported as susceptible, intermediate, or resistant. Multidrug resistance (MDR) denotes a lack of susceptibility to at least one agent in three or more chemical classes of antibiotics [18]. Antibiotic sensitivity tests conducted by the provincial hospital were solely performed on antibiotic classes used at the Thatphanom Crown Prince Hospital. Drugs that can be used for Gram-negative bacteria include quinolones and colistin, and drugs that can be used for both Gram-negative and Gram-positive bacteria include aminoglycosides, carbapenems, cephalosporins, macrolides, penicillin, and sulfonamides. Although some medications may be active against both Gram-positive bacteria and Gram-negative bacteria, MDR will be assigned regardless of drug spectrums as long as the results of AST complied with the above MDR definition.

Community-acquired infections refer to infections that are contracted external to a hospital or are diagnosed within 48 h of admission without any previous health care encounter [19].

Hospital-acquired infections refer to infections that are not present at the time of admission to a hospital and manifest after 72 h of hospitalization and with positive results of microbial culture [20].

Healthcare-associated infections refer to infections that occur while the patient receives health care for another condition at any healthcare facility. Infections in the following groups of patients are regarded as healthcare-associated infections: referred patients from other hospitals, patients who have visited any healthcare facility or dialysis clinic within the past month [19,20].

History of antibiotic use refers to a history of receiving oral antibiotics or antibiotic injections from the Thatphanom Crown Prince Hospital during the past 90 days.

History of steroid use refers to a history of receiving oral steroids or steroid injections from the Thatphanom Crown Prince Hospital during the past 90 days.

This study examined numerous variables associated with the epidemiological triad of infectious disease: a host, an external agent, and an environment [21]. Risk factors for MDR-GNB infections were chosen according to a review by Burillo et al. [22]; the selected variables were in concordance with those explored by similar research. For host factors, the selection was mainly based on the works by Falcone et al. [17] and Fernández-Martínez et al. [23] because the Thatphanom Crown Prince Hospital does not yet have guidelines for separating suspected MDR-GNB-infected patients. Selected host variables include gender [5,6], age [3,5,6,7], occupation [8], smoking history [8], alcohol history [8], medical conditions [7,8,9], site of infection [7,10], history of antibiotic use [3,6,7,8,9], history of inappropriate antibiotic use [5,8,11], and history of steroid use [5,8,11,12]. The agent variable in the context of infectious diseases is the type of pathogenic microbes that cause the disease. Four species and one group of bacteria were selected as the predictors in the current study because according to the hospital’s records, these microbes are the leading cause of death among infected patients and their prevalence is in the top ten list of high drug-resistance cases. ESBL-producing Enterobacteriaceae are any bacterial strains that produce ESBL [14,15]. Environmental variables are the conditions external to the host that cause or allow the disease to be transmitted, determine the vulnerability to infectious diseases, or increase the risk of exposure to an infectious agent. In this study, it covered the primary source of infection (community, hospital, or healthcare facility) [8,11], history of hospitalization [7,8], hospitalization period [8], hospital ward where the patient stayed during treatment [5], and usage history of any medical device that enters the body [3,5,7,8,9].

### 2.3. Statistical Analysis

Patients’ epidemiological characteristics were described using descriptive statistics: average, percentage median, and interquartile range. Risk factors for MDR-GNB were analyzed based on univariate and multivariate logistic regressions. The associations of univariate logistic regression were presented with an odds ratio (OR) and a 95% confidence interval (95% CI). Results of multivariate logistic regression were indicated as an adjusted odds ratio (OR_Adj._) and a 95% CI. This study applied a stepwise, forward variable selection technique to select variables for the final model of multivariate logistic regression, with a conventional *p*-value threshold of 0.05. A risk score for each factor of MDR-GNB infections in hospitalized patients was assigned based on its regression coefficient.

An optimal cut-off value for classification of MDR-GNB risk was determined from prediction sensitivity, specificity, and the diagnostic accuracy of each score. Receiver operating characteristic (ROC) analysis was used to calculate the prediction accuracy of the developed risk assessment tool.

### 2.4. Ethical Considerations

The study procedure and protocol were reviewed and approved by the Ethics Committee of Human Research Nakhon Phanom Provincial Health Office, Ministry of Public Health (Protocol Number REC 014/64).

## 3. Results

### 3.1. Patient Characteristics

**Figure 1:**
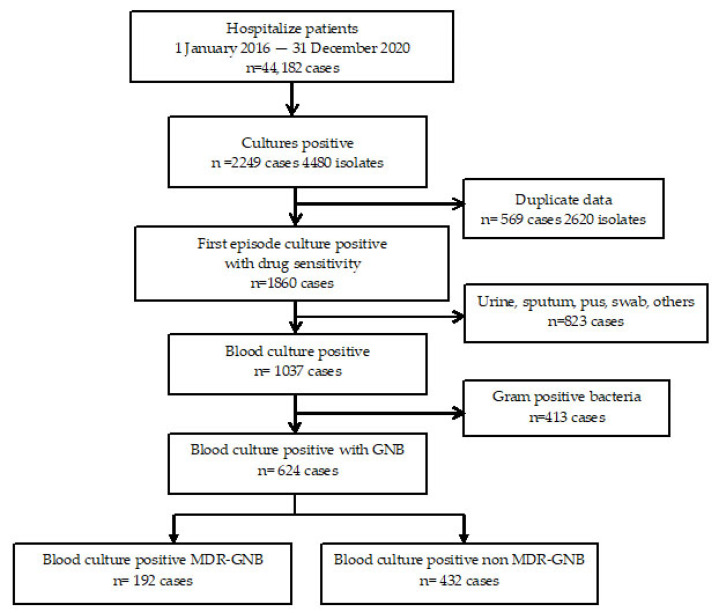
Study design and sample selection. Abbreviations: GNB, Gram-negative bacteria; MDR, multidrug resistance.

From 1 January 2016 to 31 December 2020, there were a total of 44,182 patients admitted to the community hospital in the current study. The number of specimens that tested positive for culture was 4480 isolates from 2249 patients. Among this group, 1860 patients received their first report of culture and AST. In total, 624 patients (33.55%) tested positive for GNB. The patient enrollment criteria are presented in Figure 1.

### 3.2. Characteristic of Patients with Multidrug-Resistant Gram-Negative Bacteria (MDR-GNB) Infections

The demographic characteristics of the MDR-GNB and non-MDR-GNB patients are shown in Table 1. From a total of 624 patients who tested positive for GNB, the case-to-control ratio of this study was 1:2. There were 192 patients (30.77%) who had MDR-GNB infections (cases) and 432 patients (69.23%) who had non-MDR-GNB infections (controls).

From the host perspective, the study found that most of the patients were aged over 60 years (109 cases, 56.77%), with a median age of 65 years (interquartile range ± 22.5). MDR-GNB infections were seen more often in females (107 cases, 55.73%).

This study selected bacterial type as the agent factor of infectious disease. The results of culture from specimens of cases revealed bacterial types in the following order: *Escherichia coli* (121 cases, 63.02%), *Klebsiella pneumoniae* (22 cases, 11.46%), *Burkholderia pseudomallei* (12 cases, 6.25%), and *Acinetobacter baumannii* (4 cases, 2.08%). ESBL-producing Enterobacteriaceae were positive in 28 cases (14.58%). Even though ESBL-producing Enterobacteriaceae are MDR strains, some patients may not develop MDR infections if they do not have additional risk factors. Therefore, when using logistic regression statistics to determine risk factors of MDR-GNB infections, the results showed that ESBL-producing Enterobacteriaceae were one of the factors contributing to harboring MDR-GNB at the Thatphanom Crown Prince Hospital.

Data from the environmental factors showed the source of MDR-GNB infections was largely community-acquired (183 cases, 95.31%) but not many cases had been hospitalized in the past 90 days (63 cases, 32.81%). Some cases involved previous use of any type of medical device that had entered the body in the past 90 days. Approximately half of the cases were admitted to the Thatphanom Crown Prince Hospital for at least 7 days (93 cases, 48.44%) and were generally treated in the women’s ward (84 cases, 43.75%).

### 3.3. Risk Factors for Multidrug-Resistant Gram-Negative Bacteria (MDR-GNB) Infections

#### 3.3.1. Univariate Logistic Regression Analysis

Univariate logistic regression analysis examined numerous variables associated with the epidemiological triad of infectious disease. The analysis detected a total of 10 variables as risk factors for MDR-GNB infections, whereas one factor was identified as a protective factor (Table 2).

Among all host factors being explored, only four variables were determined as risk factors for MDR-GNB infections. The most statistically significant site of infection was the urinary system (OR 2.19, 95% CI = 1.43–3.37, *p*-value < 0.001). Patients with a history of tuberculosis had a greater increased occurrence of MDR-GNB infections than those who had never had tuberculosis (OR 3.23, 95% CI 1.01–10.31, *p*-value < 0.05). The two remaining risk factors recognized during univariate logistic regression analysis were a history of steroid use in the past 90 days (OR 2.03, 95% CI 1.26–3.28, *p*-value < 0.01) and a history of smoking (OR 1.73, 95% CI 1.21–2.49, *p*-value < 0.01).

The analysis of the agent factor found that two bacterial types were associated significantly as risk factors for MDR-GNB infections. Patients developed a risk of MDR-GNB infections if they were infected with *E. coli* strains (OR 3.44, 95% CI 2.42–4.91, *p*-value < 0.001). However, they developed a very high risk of the infection with ESBL-producing Enterobacteriaceae (OR 24.41, 95% CI 7.32–81.40, *p*-value < 0.001).

Investigation of the environmental factors concluded that patients who were hospitalized during the past 90 days showed a risk of obtaining MDR-GNB infections (OR 1.56, 95% CI 1.07–2.27, *p*-value < 0.05). Furthermore, the analysis discovered that patients with some history of medical device usage in the past 90 days had a greater risk of MDR-GNB infections compared to patients with no such history. This included previous usage of a urinary catheter (OR 1.63, 95% CI 1.05–2.54, *p*-value < 0.05), endotracheal tube (OR 2.23, 95% CI 1.13–4.44, *p*-value < 0.05), or suction tube (OR 5.41, 95% CI 1.38–21.15, *p*-value < 0.05) (Table 2).

#### 3.3.2. Multivariate Logistic Regression Analysis

During multivariate logistic regression analysis, nine variables were initially selected as candidate variables. All host and environmental factors significantly associated with MDR-GNB infections were chosen. It is beneficial if a prediction tool includes a risk score of bacterial types, because once the microbial culture result arrives, additional points can be given on this topic. Only one agent factor (ESBL-producing Enterobacteriaceae) was selected for this purpose, because its OR was much higher than that of *E. coli.*

A stepwise, forward variable selection technique was used to prioritize variables toward MDR-GNB infections. It identified three important variables, including two host factors and one agent factor (Table 2). The most highly associated risk factor was the infection of ESBL-producing Enterobacteriaceae (OR 23.53, 95% CI 7.00–79.09, *p*-value < 0.001). Two host factors that contributed to a risk of MDR-GNB infections were the site of infection being the urinary system (OR 2.25, 95% CI 1.44–3.53, *p*-value < 0.001), or the patient had a history of steroid use in the past 90 days (OR 1.91, 95% CI 1.15–3.19, *p*-value < 0.05).

#### 3.3.3. Development of a Risk Assessment Tool for MDR-GNB Infections

**Figure 2:**
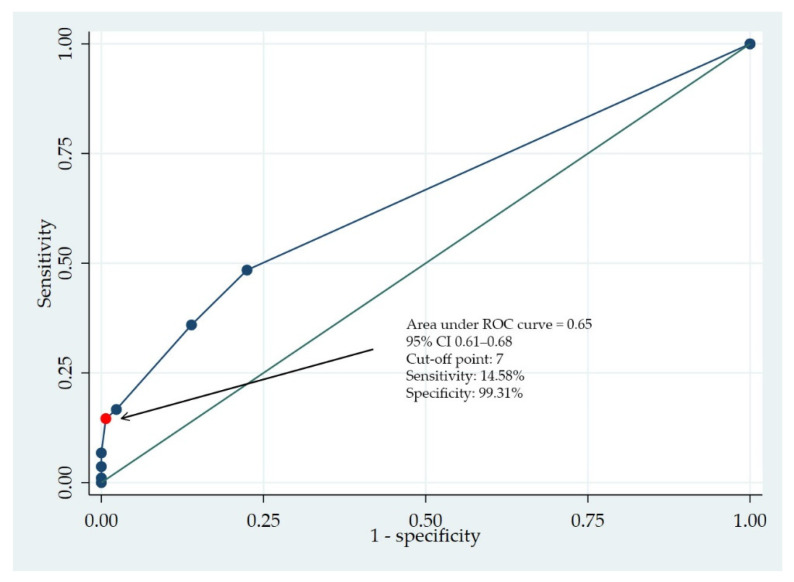
Receiver operating characteristic (ROC) analysis for prediction of MDR-GNB infections.

This study identified three risk factors for MDR-GNB infections in hospitalized patients, namely the patient having an infection of ESBL-producing Enterobacteriaceae, being diagnosed with a urinary tract infection, or having a history of steroid usage in the past 90 days. The risk assessment tool for MDR-GNB infections was developed based on these findings. This study used the area under the ROC curve (AUC-ROC) to assess the tool’s classification performance from a combined measure of sensitivity and specificity (Figure 2).

The best trade-off between the two parameters occurred at a sensitivity and specificity of 0.14 and 0.01, respectively (AUC-ROC 0.65, 95% CI 0.61–0.68). This finding indicated the tool could accurately predict the risk of MDR-GNB infection 64.54% of the time.

A statistical test for goodness of fit for the risk prediction model of MDR-GNB infections was performed using the Hosmer–Lemeshow test. The test assesses if the observed event rates match the expected event rates in population subgroups. The test returned a Hosmer–Lemeshow chi-squared value of 1.17 and a *p*-value of 0.56, indicating that this predictive model was appropriate.

Our risk assessment tool for MDR-GNB infections was equipped with a risk score for each identified risk factor, where the score was determined based on the regression coefficient of each factor. All scores were adjusted to the nearest integer so that the sum of all individual risk scores was ten points. Each GNB-positive patient received a risk score according to the risk factors he/she had. Patients who had an infection of ESBL-producing Enterobacteriaceae received a score of seven points, those diagnosed with a urinary tract infection received two points, and a score of one point was assigned to patients who had a history of steroid usage in the past 90 days (Table 3).

The risk scores of all patients who had a GNB-positive microbial culture result were grouped at different cut-off points (Table 4). At each point, the percentages of sensitivity, specificity, cases correctly classified, and the positive and negative likelihood ratios toward MDR-GNB infections were calculated.

The results indicated that using a cut-off of greater than or equal to 7 points on the assessment tool for MDR-GNB infections gave the best overall classification accuracy. This cut-off point had a sensitivity of 14.58% and a specificity of 99.31%, which indicated that 14.58% of the patients were correctly classified as having a “MDR-GNB infection” and 99.31% as having a “non-MDR-GNB infection”. The percentage of cases correctly classified depicts the ability of a tool to state that patients who test positive are more likely to develop the disease and those who test negative are less likely to have the disease. The percentage of cases correctly classified was maximum (73.24%) at a cut-off of greater than or equal to 7 points. Data from positive and negative likelihood ratio analyses (LR+, LR−) indicated that at this cut-off point, the likelihood that the prediction will be true-positive instead of false-positive was 21, whereas the likelihood that it was a true-negative instead of false-negative was 0.86. Based on the values of LR+ and LR−, the results indicated that a cut-off point of greater than or equal to 7 for risk assessment of MDR-GNB infections was often conclusive in determining true MDR-GNB cases (LR+ 21.00). In addition, this cut-off point had a low LR− of 0.86, which indicated that the tool had a greater ability to differentiate people with the disease from people without the disease. In summary, a risk score of greater than or equal to 7 points was chosen as the cut-off point to determine patients at risk of MDR-GNB infections.

## 4. Discussion

Our study of risk factors for MDR-GNB infections at the Thatphanom Crown Prince Hospital using univariate and multivariate logistic regression analyses identified three significantly associated variables: ESBL-producing Enterobacteriaceae infection (OR_Adj._ 23.53, 95% CI 7.00–79.09, *p*-value < 0.001), urinary tract infection (OR_Adj._ 2.25, 95% CI 1.44–3.53, *p*-value < 0.001), and a history of steroid use within the past 90 days (OR_Adj._ 1.91, 95% CI 1.15–3.19, *p*-value < 0.05).

The greatest associated risk factor discovered by the study was ESBL-producing Enterobacteriaceae infection. Patients who were infected with this bacterial type had a 23.53 times increased risk of MDR-GNB infections compared to patients without the pathogen. This finding was in concordance with some national retrospective cohort studies [24,25]. A prediction model for MDR infections reported infection of ESBL-producing Enterobacteriaceae as one of the 12 risk factors (OR_Adj._ 5.77, 95% CI 4.04–8.23, *p*-value < 0.0001) [24]. Another implementation created to predict whether a bacteremia patient was infected with an ESBL-producing organism suggested five risk factors, among them being a history of infection with ESBL-producing bacteria (OR_Adj._ 50.68, 95% CI 25.97–98.92) [25].

A previous study conducted in hospitals of Nakhon Phanom province demonstrated that prior cephalosporins therapy was associated with infections from ESBL-producing *E. coli* or *K. pneumoniae* in hospitalized patients [26]. The Thatphanom Crown Prince Hospital estimated that ceftriaxone consumption was likely to increase every year [27]; therefore, usage of these medications should be properly monitored to reduce future MDR incidence.

The current study specified the most important site of infection producing MDR-GNB being the urinary system. The predictive model from our study was consistent with two retrospective cohort studies that examined risks of MDR-GNB from all specimen types [24]. A risk prediction model by González Del Castillo J et al. reported that UTI increased the chance of MDR-GNB infections (OR 1.38, 95% CI 1.01–1.88, *p*-value = 0.043) [24]. Another publication investigated the predictive factors of MDR-GNB infections in patients with complicated UTIs. The multicenter study discovered five risk factors, including having UTI within the previous year (OR 1.89, 95% CI 1.28–2.79, *p*-value = 0.001) [28].

A few studies did not recognize UTI as a key variable of MDR-GNB infections; hence, UTI was not listed in their predictive models [29]. A model established by Zilberman-Itskovich et al. listed 10 risk factors for MDR infections but it did not incorporate UTI, possibly because the study addressed a much wider range of possible causes and UTI did not demonstrate a strong influence toward MDR infections [29]. A retrospective cohort study by Patolia S. et al. indicated the urinary catheter as a source of infection that promoted the development of MDR-GNB (OR 5.96, 95% CI 1.78–19.94, *p*-value < 0.05) and increased the mortality in patients with MDR-GNB bacteremia (OR 5.6, 95% CI 1.37–23.5, *p*-value < 0.05) [30]. Notably, UTI was not a risk factor despite the fact that the authors investigated this variable. A possible reason that differentiated their results from the current study was the choice of research setting. The previous authors conducted their study in a liver transplant and cancer center, while the current study was performed in a community hospital; therefore, the prevalence of infection types that were seen in cases and controls could be different. Nonetheless, from our perspective, using a urinary catheter makes the patients more susceptible to urinary tract infections. According to a report from the Thatphanom Crown Prince Hospital during 2019–2020, the most common type of infection among hospitalized patients was urinary tract infection [31].

The conclusion from the current study that the risk of MDR-GNB infections increased if the patient used steroids in the past 90 days was quite reasonable because such medication weakens the immune system. Research in a cancer hospital in Thailand published similar results to this, stating that using steroids was a risk factor for MDR [32]. Similarly, a retrospective study by Viasus D. et al. demonstrated the negative effect of steroids on MDR, since it increased the chance of developing MDR-*Pseudomonas aeruginosa* infections in neutropenic patients by 2.92 times (OR 2.92, 95% CI 1.15–7.40) [33]. Notably, although the current study agreed with that of Gomila A et al. on the role of UTI toward MDR-GNB infection, the inference on the risk of steroid usage differs [28]. The previous authors did not find steroid usage or immunosuppressive therapy as predictive factors for MDR-GNB risk [28].

The current study has proposed a newly developed risk assessment tool for MDR-GNB infections, comprising three highly associated variables. Each factor was assigned a risk score according to its regression coefficient from multivariate analysis. The highest possible score per patient was 10 and a cut-off point of ≥7 was used to differentiate patients who were likely to have MDR-GNB infections. ROC analysis indicated an AUC of 0.65 (95% CI 0.61–0.68), indicating that the tool has a prediction accuracy of 64.54% (95% CI 60.69–68.33). Earlier tools that stratified patients who were at risk of having MDR or MDR-GNB infections established a predictive performance of 70–96% [17,23,29,34,35]. The variations may have been caused by differences in the size of the hospital where the research was conducted, the number of cases and controls, the number of data being collected per patient, the number of candidate variables identified during initial analysis, and the number of risk factors in the final predictive model. Effective implementation of a risk assessment tool for practical use requires consideration of where the tool is intended to be used and whether the evaluating data can be accessed easily and quickly. A tool that requires a very specific dataset is not suitable for everyday use in community hospitals.

Because each tool is developed according to a unique study design, clearly the overall prediction performance may differ among them. Using 3 predictive components as opposed to the 5–10 components of other studies, our tool had an acceptable performance of 64.54% in classifying patients with high and low risks of MDR-GNB infections. An earlier study that produced a tool with 70.00% prediction accuracy (95% CI 63.40–75.60) used a total of four predictive risk factors to evaluate the risk of having MDR-GNB in patients admitted to intensive care units [23]. The model with the best predictive performance for MDR-GNB was developed with nine predictive variables; it successfully categorized 75.00% (95% CI 69.00–81.00) of patients as having high or low risk of MDR-GNB infection [35].

A tool’s sensitivity and specificity are inversely proportional—the higher the sensitivity, the lower the specificity. Our tool had 14.58% sensitivity and 99.31% specificity. It was comparable to an MDR-GNB prediction tool developed by Fernández-Martínez et al. that produced a sensitivity of 5.5% and a specificity of 99.90% [23], and to an MDR assessment tool that showed a sensitivity of 82.20% and specificity of 95.70% [34].

For MDR-GNB prediction, sensitivity describes the tool’s performance to correctly classify “MDR-GNB infection” in a patient. A tool with high sensitivity promotes prompt identification of a patient who is likely to have an MDR-GNB infection. High-risk patients should be isolated to reduce the spread of MDR-GNB to other patients. Specificity represents the tool’s ability to correctly classify “non-MDR-GNB infection”. If a tool has a higher specificity, its sensitivity drops. This creates further misclassifications that then force the isolation of patients who do not have a high risk for MDR-GNB. Such a condition leads to an unnecessarily increased budget for the care of high risk MDR-GNB patients. Therefore, based on our results, the tool gave a sensible trade-off between sensitivity and specificity when the classification cut-off was set at greater than or equal 7 points.

When a patient’s risk score is greater than or equal to 7 points, the ward nurse should take action to prevent and control the spread of drug-resistant bacteria using the guidelines for the Infection Prevention and Control [36]. The nurse should create an alert in the hospital’s HOSxP program to specify which patient requires surveillance for MDR-GNB infection and transmission. The alert also aims to inform physicians to be aware of antibiotic selection and notify pharmacists to control and review the medication and ensure it is properly prescribed. This implementation will be one of the strategies to reduce antimicrobial resistance because it can promote the appropriate use of antimicrobial agents. Reducing antibiotic resistance would mean a reduction in the cost of medical care for resistant strains.

In summary, our 5-year retrospective case–control study collected data on the blood culture results and epidemiological characteristics of patients admitted to the Thatphanom Crown Prince Hospital. This was the first research article to investigate the risks of MDR-GNB infections at this facility. Due to the nature of the retrospective study, some of the variables that may need to be considered could not be included, such as the fact that some patients had been admitted to this hospital for the first time, making it impossible to obtain their comprehensive medical history that had been recorded by other facilities. Nevertheless, the tool demonstrated that it can be used to assess GNB infection cases in a community hospital to promote practical guidance on MDR-GNB evaluation and prevention. Future studies should include the incorporation and evaluation of risk factors identified from other studies into our model and the continuous surveying of antibiotic-resistant situations in hospital to update the risk factors of MDR-GNB infections. These plans should promote outstanding practice in antimicrobial stewardship programs in Thailand’s community hospitals.

These findings highlight the importance of considering patient factors such as site of infection—urinary system, drug factor—steroid usage in past 90 days, and agent factors—ESBL-producing Enterobacteriaceae. Specifically, there are three variables for the risk score assessment tool for MDR-GNB infections. Lastly, the odds ratio of the logistic regression model was low, indicating that further prospective research is urgently needed to explore additional factors that may be used for antibiotic treatment, comorbid conditions, transfers from long-term care facilities, and other agent factors.

The limitations for our study include the following factors: single-center study, retrospective analysis of clinical data, relatively smaller sample size, and small number of patients with risk factors for MDR-GNB. Therefore, the effects of OR on the three risk factors for MDR-GNB were developed for a risk assessment tool for MDR-GNB infections. The AUC-ROC used to assess the tool was the area under the ROC curve. Furthermore, the tool could accurately predict the risk prediction model of MDR-GNB and the Hosmer–Lemeshow test, indicating that the predictive model was appropriate but not significant. In addition, the monitoring in the hospital should be considered a risk factor that is significant with univariate analysis.

## 5. Conclusions

This study utilized a dataset compiled from 5 years of retrospective case–control data to determine the risk factors of MDR-GNB infections in a middle-sized community hospital. Numerous variables from GNB-positive patients were analyzed and key findings were incorporated into a risk assessment tool for MDR-GNB infections. The tool comprises three risk factors; therefore, it can be easily used to classify hospitalized patients. The presence of high-risk MDR-GNB patients in a hospital requires an immediate alert in the hospital system, so it is best if these patients can be predicted accurately and promptly. Our predictive tool supports the antibiotic stewardship program since it provides a method for rapid patient evaluation and suggests what action should be taken to prevent and control the spread of MDR-GNB to other hospitalized patients.

In conclusion, the risk factors for infections due to multidrug-resistant Gram-negative bacteria in a community hospital in rural Thailand were as follows: eleven factors with univariate logistic regression and three factors with multivariate logistic regression. In practice, monitoring in hospitals should consider the overall risk factors from recommended guidelines for MDR-GNB. The risk assessment tool for MDR-GNB infections was moderately predictive. The effect of the three factors and the small sample size were used to the predict the value of AUC-ROC. Further studies should explore the risk factors and study multicenter studies in small–medium community hospitals.

## Figures and Tables

**Table 1:** Patients’ epidemiological characteristics with blood culture results that were GNB-positive (*n* = 624).

Characteristic	Non-MDR-GNB (Total 432 Patients) *n* (%)	MDR-GNB (Total 192 Patients) *n* (%)
HOST FACTORS		
Age		
<60 years	212 (49.07)	83 (43.23)
≥60 years	220 (50.93)	109 (56.77)
Median ± interquartile range (years)	60 ± 24	65 ± 22.5
Gender		
Male	214 (49.54)	85 (44.27)
Female	218 (50.46)	107 (55.73)
Occupation		
Agriculture	241 (55.79)	105 (54.69)
Unemployed	123 (28.47)	54 (28.13)
Employee	42 (9.72)	18 (9.38)
Merchant	11 (2.55)	4 (2.08)
Government officer	10 (2.31)	9 (4.69)
Priest	5 (1.16)	2 (1.04)
Smoking history	113 (26.16)	73 (38.02)
Drinking history	173 (40.05)	90 (46.88)
Underlying disease		
Diabetes mellitus	89 (20.60)	43 (22.40)
Hypertension	65 (15.05)	31 (16.15)
Chronic renal failure	14 (3.24)	6 (3.13)
Chronic obstructive pulmonary disease (COPD)	14 (3.24)	2 (1.04)
Cerebrovascular accident (CVA)	6 (1.39)	2 (1.04)
Acquired Immunodeficiency Syndrome (AIDS)	3 (0.69)	4 (2.08)
Past illness		
Tuberculosis (TB)	5 (1.16)	7 (3.65)
Site of infection		
Urinary system	57 (13.19)	48 (25.00)
Respiratory system	70 (16.20)	28 (14.58)
Wound	12 (2.78)	9 (4.69)
Blood	187 (43.29)	68 (35.42)
Drug factor		
Antibiotics usage in past 90 days	16 (3.70)	14 (7.29)
Inappropriate antibiotics usage in past 90 days	12 (2.78)	11 (5.73)
Steroid usage in past 90 days	44 (10.19)	36 (18.75)
AGENT FACTORS		
*Escherichia coli*	143 (33.10)	121 (63.02)
*Burkholderia pseudomallei*	89 (20.60)	12 (6.25)
*Klebsiella pneumoniae*	56 (12.96)	22 (11.46)
*Acinetobacter baumannii*	18 (4.17)	4 (2.08)
ESBL-producing Enterobacteriaceae	3 (0.69)	28 (14.58)
ENVIRONMENT FACTORS		
Source of infection		
Community-acquired infection	423 (97.92)	183 (95.31)
Hospitalized in past 90 days	103 (23.84)	63 (32.81)
Medical device usage in past 90 days		
Heparin lock	92 (21.30)	48 (25.00)
Urinary catheter	60 (13.89)	40 (20.83)
Nasogastric tube	14 (3.24)	6 (3.13)
Suction tube	3 (0.69)	7 (3.65)
Endotracheal tube	18 (4.17)	17 (8.85)
Length of hospitalization		
≥7 days	182 (42.13)	93 (48.44)
Ward		
Female ward	157 (36.34)	84 (43.75)
Male ward	154 (35.65)	73 (38.02)
Special ward	67 (15.51)	24 (12.50)
Pediatric ward	30 (6.94)	4 (2.08)
Intensive care unit	24 (5.56)	7 (3.65)

**Table 2:** Assessment of risk factors for MDR-GNB infections using univariate and multivariate logistic regression analyses (*n* = 624).

Risk Factor	Univariate		Multivariate	
	OR (95% CI)	*p*-Value	OR_Adj._ (95% CI)	*p*-Value
HOST FACTORS				
Age				
<60 years	0.80 (0.56–1.11)			
≥60 years	1.27 (0.90–1.78)	0.177		
Gender				
Male	0.81 (0.58–1.14)	0.225		
Female	1.24 (0.88–1.74)			
Occupation				
Agriculture	0.96 (0.68–1.35)	0.799		
Unemployed	0.98 (0.67–1.43)	0.929		
Employee	0.96 (0.54–1.72)	0.892		
Merchant	0.81 (0.26–2.59)	0.728		
Government officer	2.08 (0.83–5.19)	0.119		
Priest	0.90 (0.17–4.67)	0.899		
Smoking history	1.73 * (1.21–2.49) ^a^	0.003		
Drinking history	1.32 (0.94–1.86)	0.111		
Underlying disease				
Diabetes Mellitus	1.11 (0.74–1.68)	0.613		
Hypertension	1.09 (0.68–1.73)	0.725		
Chronic renal failure	0.96 (0.36–2.55)	0.940		
Chronic obstructive pulmonary disease (COPD)	0.31 (0.07–1.40)	0.128		
Cerebrovascular accident (CVA)	0.75 (0.15–3.74)	0.723		
Acquired Immunodeficiency Syndrome (AIDS)	3.04 (0.67–13.73)	0.148		
Past illness				
Tuberculosis (TB)	3.23 * (1.01–10.31) ^a^	0.048		
Site of infection				
Urinary system	2.19 ** (1.43–3.37) ^a^	<0.001	2.25 ** (1.44–3.53)	<0.001
Respiratory system	0.31 (0.07–1.40)	0.128		
Wound	1.72 (0.71–4.16)	0.227		
Blood	0.72 (0.51–1.02)	0.065		
Drug factor				
Antibiotics usage in past 90 days	2.04 (0.98–4.28)	0.058		
Inappropriate antibiotics usage in past 90 days	2.13 (0.92–4.91)	0.077		
Steroid usage in past 90 days	2.03 * (1.26–3.28) ^a^	0.004	1.91 * (1.15–3.19)	0.013
AGENT FACTORS				
*Escherichia coli*	3.44 ** (2.42–4.91)	<0.001		
*Burkholderia pseudomallei*	0.26 ** (0.14–0.48)	<0.001		
*Klebsiella pneumoniae*	0.87 (0.51–1.47)	0.600		
*Acinetobacter baumannii*	0.49 (0.16–1.47)	0.202		
ESBL-producing Enterobacteriaceae	24.41 ** (7.32–81.40) ^a^	<0.001	23.53 ** (7.00–79.09)	<0.001
ENVIRONMENT FACTORS				
Source of infection				
Community-acquired infection	0.43 (0.17–1.11)	0.081		
Hospitalization in past 90 day	1.56 * (1.07–2.27) ^a^	0.020		
Medical devices usage in past 90 day				
Heparin lock	1.23 (0.83–1.84)	0.306		
Urinary catheter	1.63 * (1.05–2.54) ^a^	0.030		
Nasogastric tube	0.96 (0.36–2.55)	0.940		
Suction tube	5.41 * (1.38–21.15) ^a^	0.015		
Endotracheal tube	2.23 * (1.13–4.44) ^a^	0.022		
Length of hospitalization				
<7 days	0.77 (0.55–1.09)	0.143		
≥7 days	1.29 (0.92–1.82)			
Ward				
Female ward	1.36 (0.96–1.93)	0.080		
Male ward	1.11 (0.78–1.57)	0.570		
Special ward	0.78 (0.47–1.28)	0.320		
Pediatric ward	0.29 * (0.10–0.82)	0.020		
Intensive care unit	0.64 (0.27–1.52)	0.314		

* *p*-value < 0.05, ** *p*-value < 0.001, ^a^ Variables initially used during multivariate logistic regression analysis.

**Table 3:** Score assignment for risk factors of MDR-GNB infections.

Risk Factor	Score
ESBL-producing Enterobacteriaceae	7
Urinary tract infection	2
Steroid usage in past 90 days	1

**Table 4:** Cut-off point determination and prediction accuracy of risk assessment tool for MDR-GNB infections.

Cut-Off Point	Sensitivity (%)	Specificity (%)	Correctly Classified (%)	LR+	LR−
≥0	100.00	0.00	30.77	1.00	
≥1	48.44	77.55	68.59	2.16	0.66
≥2	35.94	86.11	70.67	2.59	0.74
≥3	16.67	97.69	72.76	7.20	0.85
≥7	14.58	99.31	73.24	21.00	0.86
≥8	6.77	100.00	71.31		0.93
≥9	3.65	100.00	70.35		0.96
≥10	1.04	100.00	69.55		0.98

Abbreviations: LR+, positive likelihood ratio; LR−, negative likelihood ratio.

## Data Availability

The datasets used and/or analyzed during the current study are available from the corresponding author upon reasonable request.
